# Supplementary material for: Neurotoxic amyloidogenic peptides in the proteome of SARS-COV2: potential implications for neurological symptoms in COVID-19
Source: Nat Commun. 2022 Jun 13;13:3387. doi: 10.1038/s41467-022-30932-1 (PMC9189797; doi:10.1038/s41467-022-30932-1)
Supplement: Supplementary file 1 — Supplementary Information [file 41467_2022_30932_MOESM1_ESM.pdf]

Neurotoxic Amyloidogenic Peptides in the Proteome of SARS-COV2: Potential Implications for Neurological Symptoms in COVID-19

Mirren Charnley[1,2], Saba Islam[3], Guneet Bindra[3], Jeremy Engwirda[3], Julian Ratcliffe[4], Jiangtao Zhou[5], Raffaele Mezzenga[5], Mark Hulett[3], Kyunghoon Han[6], Joshua T. Berryman[6],*, and Nicholas P. Reynolds[3],*

[1]Centre for Optical Sciences and Department of Health Sciences and Biostatistics, Swinburne University of Technology, Hawthorn, Victoria 3122, Australia

[2]Immune Signalling Laboratory, Peter MacCallum Cancer Centre, Parkville, Victoria, 3000, Australia

[3]Department of Biochemistry & Chemistry, La Trobe Institute for Molecular Science, La Trobe University, Bundoora, Victoria, 3086, Australia

[4]La Trobe University Bioimaging Platform, Bundoora 3086, Victoria, Australia

[5]ETH Zurich, Department of Health Sciences & Technology, Schmelzbergstrasse 9, LFO, E23, 8092 Zurich, Switzerland

[6]Department of Physics and Materials Science, Faculty of Science, Technology and Medicine, University of Luxembourg, 162a Avenue de la Faïencerie, L-1511 Luxembourg

Supplementary Data

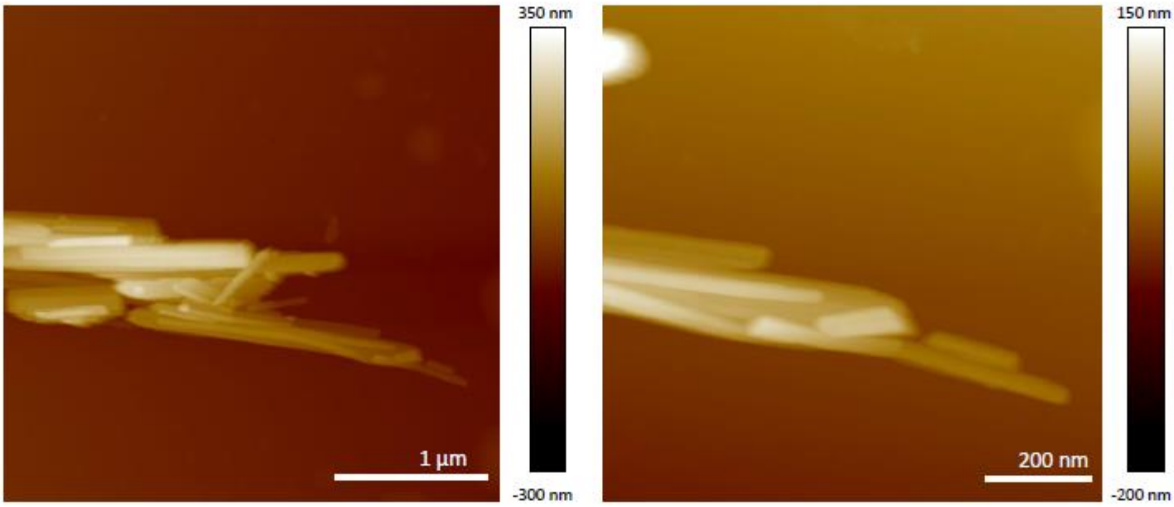

Supplementary Figure 1: AFM Images of RNYIAQVD assemblies [1 mg mL^{-1}] after 2 h assembly. All AFM images are taken at 512 x 512 scan lines x pixels, at a scanning speed of 0.5 Hz displayed at a z-height of 40 nm using a black-golden-white colour gradient. Images are representative images selected from three independent experiments.

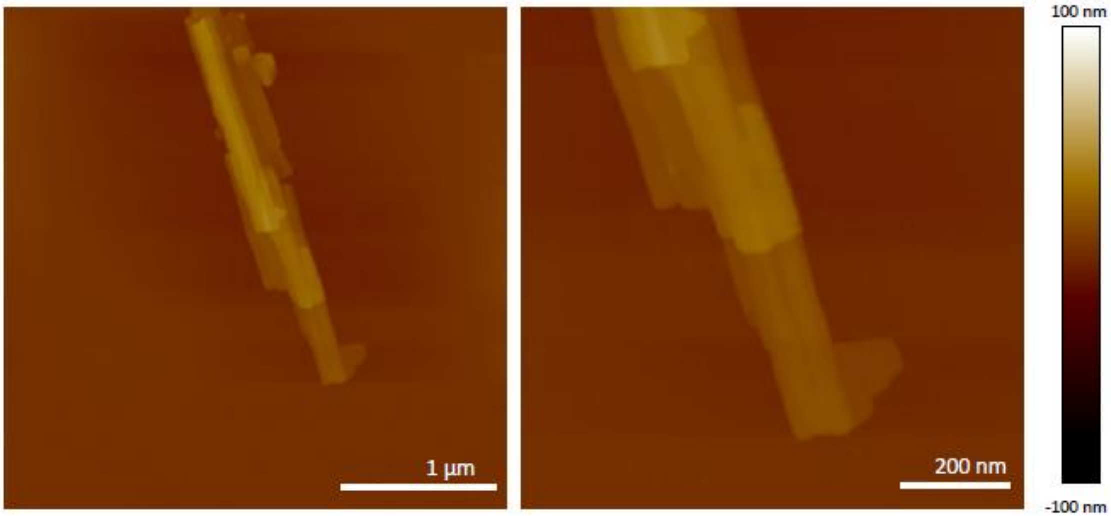

Supplementary Figure 2: AFM Image of ILLIIM assemblies [1 mg mL^{-1}] after 2 h assembly. all AFM images are taken at 512 x 512 scan lines x pixels, at a scanning speed of 0.5 Hz displayed at a z-height of 40 nm using a black-golden-white colour gradient. Images are representative images selected from three independent experiments.

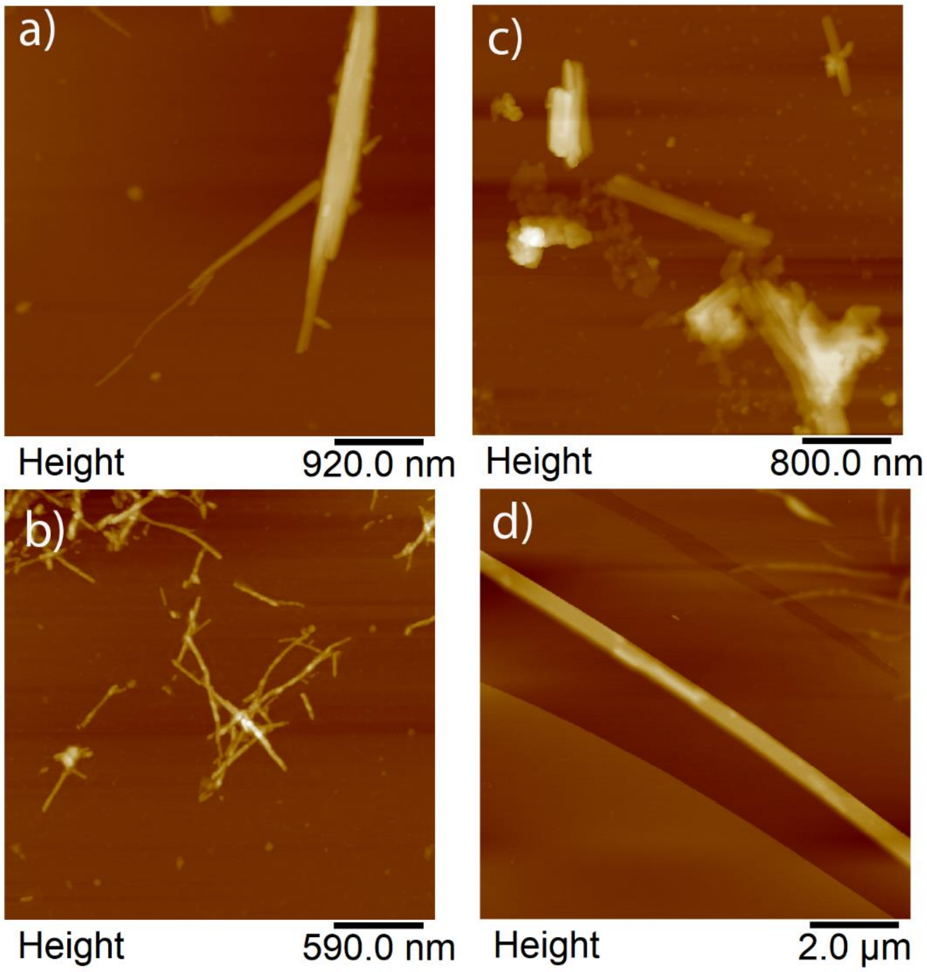

Supplementary Figure 3: AFM images showing greater polymorphism occurring with self-assembly at 37 ºC (5 mg mL^{-1}). RNYIAQVD (a) needle-like crystals and (b) nanofibrils. (c,d) Different crystalline polymorphs of ILLIIM. NB. At all assembly conditions tested the crystalline needle polymorph was the dominant one for both peptides. All AFM images are taken at 512 x 512 scan lines x pixels, at a scanning speed of 0.5 Hz displayed at a z-height of 40 nm using a black-golden-white colour gradient. Images are representative images selected from three independent experiments.

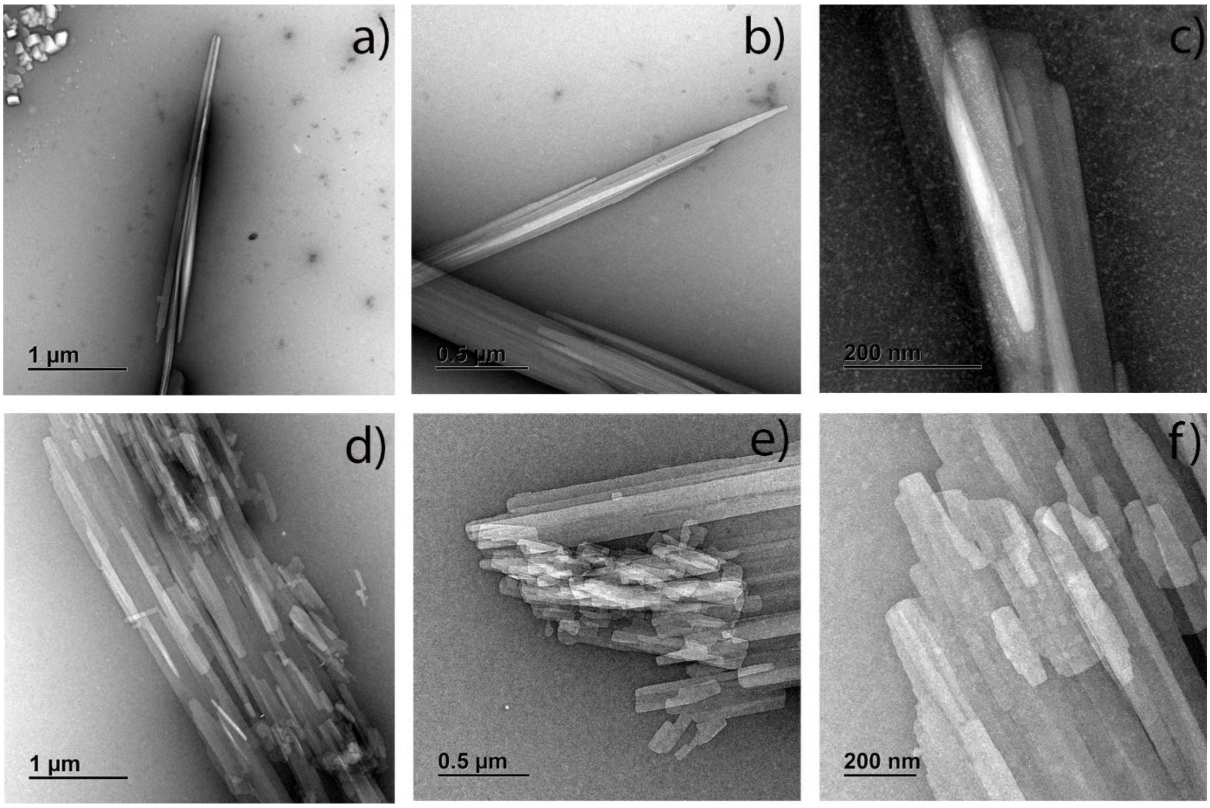

Supplementary Figure 4: Additional TEM images of ORF6 & ORF10 peptide assemblies a-c) RNYIAQVD d-f) ILLIIM (24 h assembly, slow cooling method). Images are representative images selected from three independent experiments.

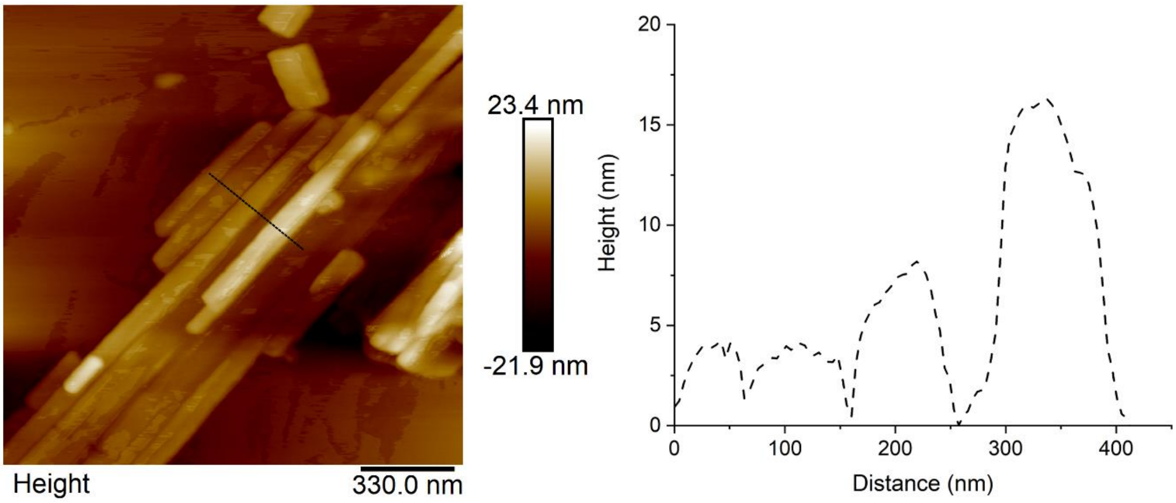

Supplementary Figure 5: Line scans of ILLIIM assemblies (5 mg mL^{-1}) (24 h assembly). All AFM images are taken at 512 x 512 scan lines x pixels, at a scanning speed of 0.5 Hz displayed at a z-height of 40 nm using a black-golden-white colour gradient. Images are representative images selected from three independent experiments.

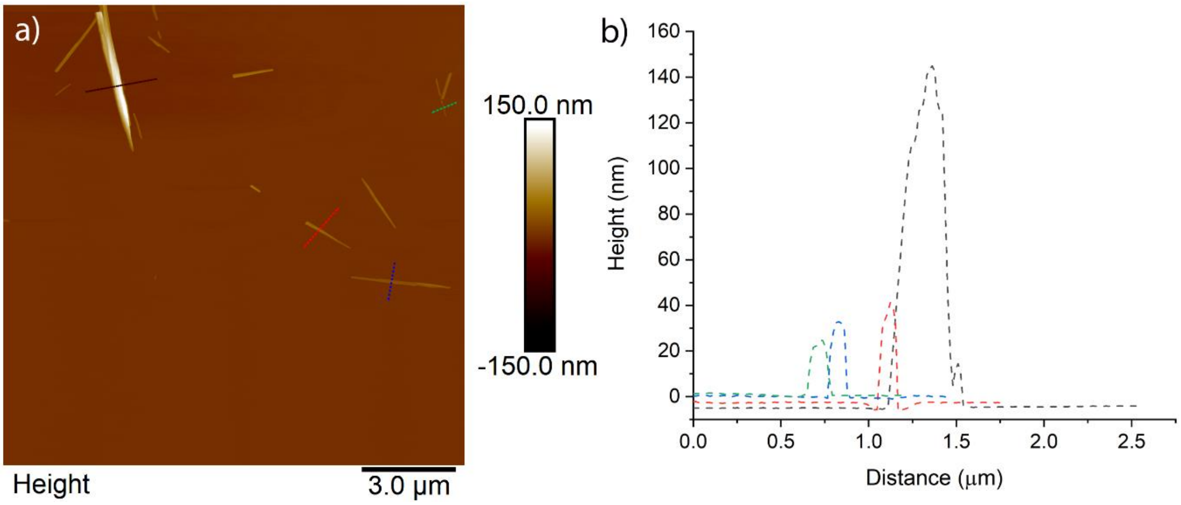

Supplementary Figure 6: Line scans of RNYIAQVD assemblies (5 mg mL^{-1}) (24 h assembly). all AFM images are taken at 512 x 512 scan lines x pixels, at a scanning speed of 0.5 Hz displayed at a z-height of 40 nm using a black-golden-white colour gradient. Images are representative images selected from three independent experiments.

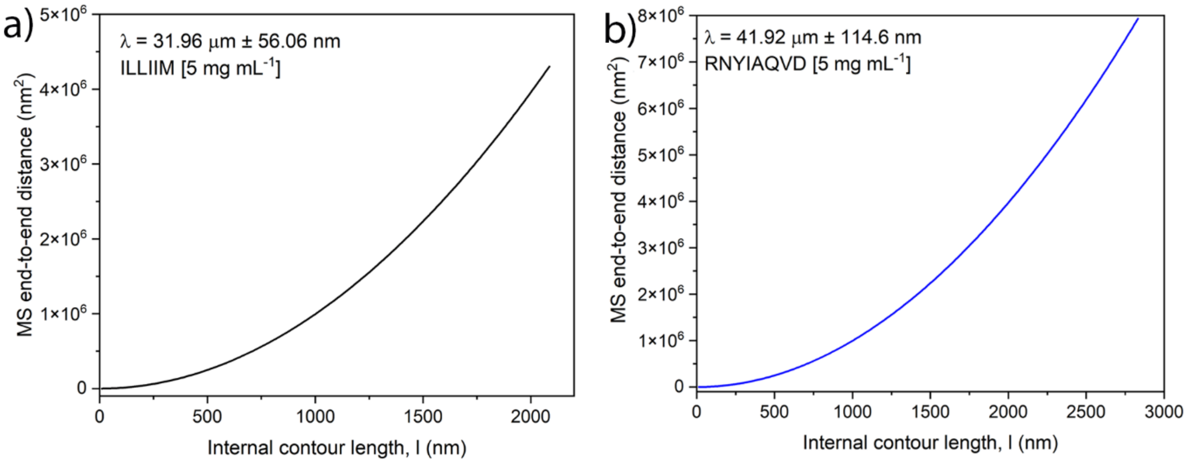

Supplementary Figure 7: Mean-Square end-to-end persistence length calculations for both peptide assemblies

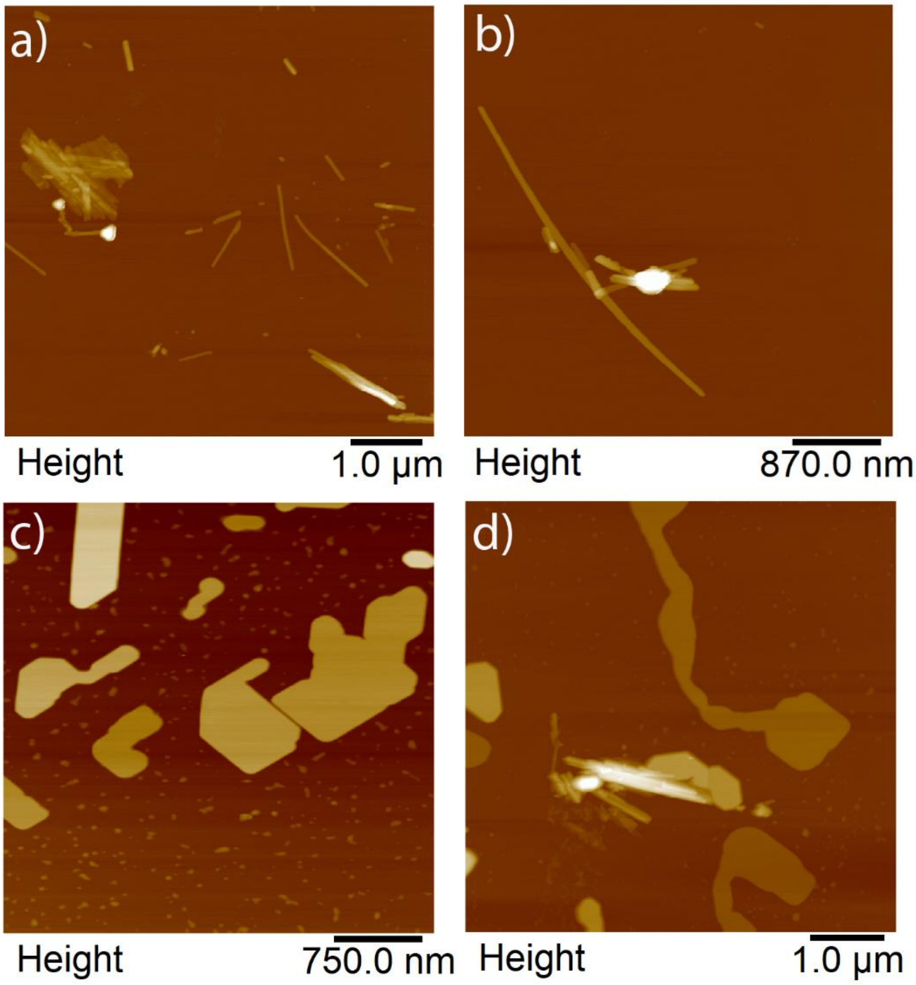

Supplementary Figure 8: Evidence of co-crystallisation of ILLIIM and RNYIAQVD peptides. AFM images showing a wide range of polymorphic crystalline, fibrous and amorphous assemblies including those never observed with single peptide assemblies. All images are 512 x 512 pixels, taken at a scan speed of < 1Hz, z-ranges were adjusted for optimal contrast (a) z-range = 160 nm, (b) z-range = 200 nm, c) z-range = 80 nm, d) z-range = 300 nm. All AFM images are taken at 512 x 512 scan lines x pixels, at a scanning speed of 0.5 Hz using a black-golden-white colour gradient. Images are representative images selected from three independent experiments.

	Left Twisted β-sheet (%)	Right Twisted β-sheet (%)	Relaxed β-sheet (%)	β-turn (%)	α-helix (%)	Unclassified (%)
RNYIAQVD 5 mg mL^{-1}	0	49.6	0	0	9.2	41.2
ILLIIM 5 mg mL^{-1}	41.8	0	0	58.2	0	0
RNYIAQVD 1 mg mL^{-1}	0	38.1	0	0	18.8	43.1
ILLIIM 1 mg mL^{-1}	17.1	19.4	32.1	31.4	0	0

Supplementary Table 1: Secondary Structure Analysis of CD spectra

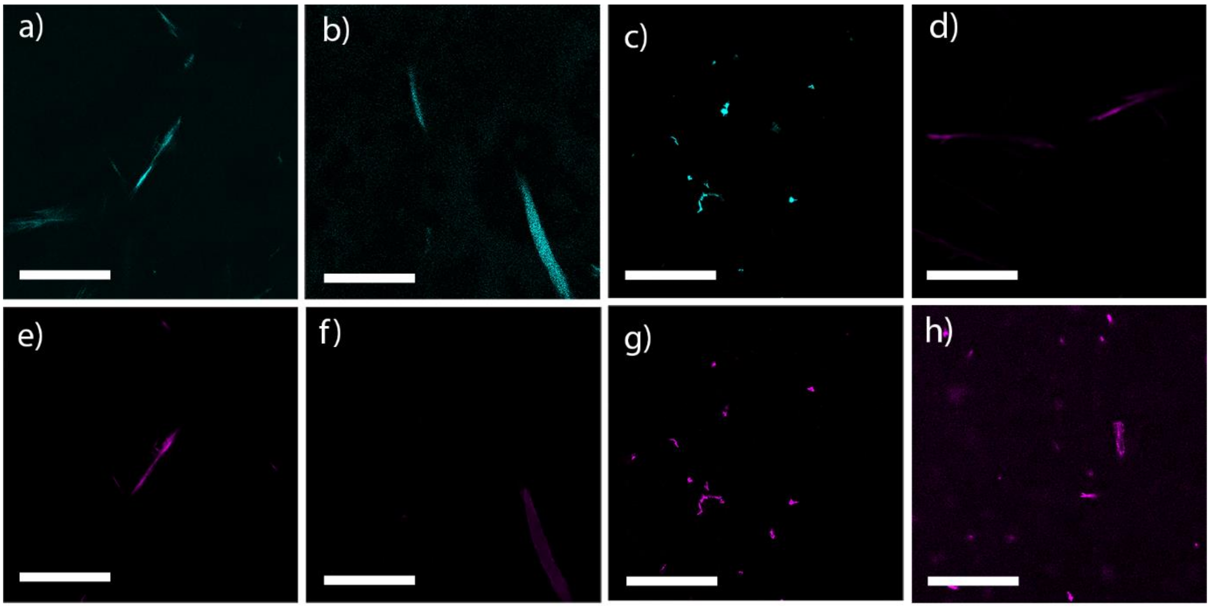

Supplementary Figure 9: ThT (top) and Oligomer Staining (A11 antibody) (bottom) immunofluorescence micrographs of ILLIIM and RNYIAQVD assemblies (5 mg mL^{-1}). a) ThT staining of RNYIAQVD, b) ThT staining of ILLIIM, c) ThT staining of phenylalanine oligomers (positive control), d) 2^0 antibody only staining of ILLIIM (negative control) mean fluorescent intensity = 31.14 ± 7.17 a.u, e) A11 staining of RNYIAQVD mean fluorescent intensity = 35.45 ± 9.54 a.u, f) A11 staining of ILLIIM mean fluorescent intensity = 30.62 ± 6.87 a.u, g) A11 staining of phenylalanine oligomers (positive control) mean fluorescent intensity = 147.74 ± 13.19 a.u, h) 2^0 antibody only staining of RNYIAQVD (negative control) mean fluorescent intensity = 45.47 ± 6.74 a.u. All scale bars = 10 µm. No A11 positive staining for any panels except the positive control suggest negligible oligomer formation at this assembly point. Mean fluorescence intensity ± sem calculated from at least 10 different assemblies. Images selected from three experiments.

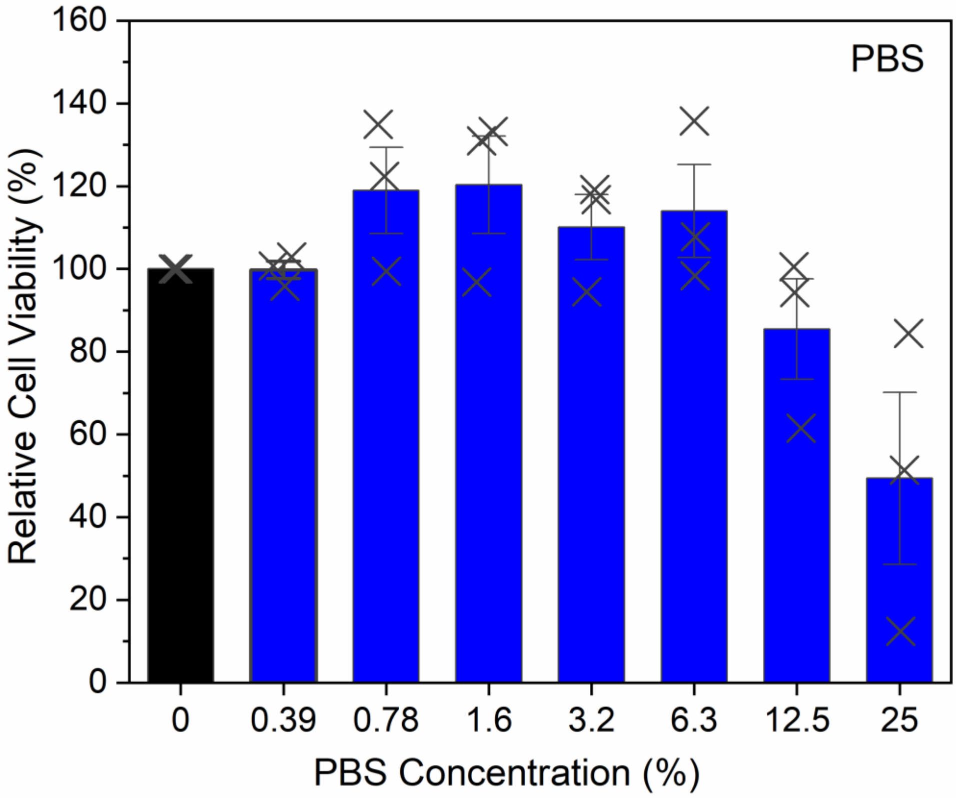

Supplementary Figure 10: Cytotoxicity assays showing the toxicity of PBS in the concentrations used for peptide cytotoxicity assays. Data are presented as mean values +/- SEM of mean of three independent experiments (n=3).

**Figure 5a**				**Figure 5b**		
ILLIIM				RNYIAQVD		
Comparison (mg/mL)	Statistically Significant	P = Value		Comparison (mg/mL)	Statistically Significant	P = Value
0 vs 0.04	***	<0.0001		0 vs 0.04	ns	0.0651
0 vs 0.08	***	<0.0001		0 vs 0.08	***	0.0004
0 vs 0.15	***	<0.0001		0 vs 0.15	***	<0.0001
0 vs 0.3	***	<0.0001		0 vs 0.3	***	<0.0001
0 vs 0.6	***	<0.0001		0 vs 0.6	***	<0.0001
0 vs 1.25	***	<0.0001		0 vs 1.25	***	<0.0001
0 vs 2.5	***	<0.0001		0 vs 2.5	***	<0.0001
**Figure 5d**				**Figure 5e**		
ILLIIM				RNYIAQVD		
Comparison (mg/mL)	Statistically Significant	P = Value		Comparison (mg/mL)	Statistically Significant	P = Value
0 vs 0.02	ns	0.5403		0 vs 0.02	ns	0.9976
0 vs 0.04	**	0.0011		0 vs 0.04	ns	0.8331
0 vs 0.08	**	0.0069		0 vs 0.08	ns	0.2554
0 vs 0.15	ns	0.0661		0 vs 0.15	*	0.0356

0 vs 0.3	**	0.008		0 vs 0.3	ns	0.1927
0 vs 0.6	**	0.0068		0 vs 0.6	**	0.0062
0 vs 1.25	**	0.0025		0 vs 1.25	***	0.0001
0 vs 2.5	**	0.0011		0 vs 2.5	****	<0.0001
**Figure 5f**				**Figure 5g**		
ILLIIM				RNYIAQVD		
Comparison (mg/mL)	Statistically Significant	P = Value		Comparison (mg/mL)	Statistically Significant	P = Value
0 vs 0.02	ns	0.1104		0 vs 0.02	ns	0.1519
0 vs 0.04	**	0.0045		0 vs 0.04	ns	0.2735
0 vs 0.08	ns	0.3161		0 vs 0.08	ns	0.0893
0 vs 0.15	**	0.0055		0 vs 0.15	*	0.0338
0 vs 0.3	*	0.0266		0 vs 0.3	ns	0.0706
0 vs 0.6	*	0.0181		0 vs 0.6	ns	0.057
0 vs 1.25	**	0.0055		0 vs 1.25	*	0.0172
0 vs 2.5	**	0.0024		0 vs 2.5	*	0.015

Supplementary Table 2: P-Values for the statistical analysis performed in figure 5

Gating Strategy for Flow Cytometry

Boundaries between viable (negative 7-AAD staining, lower quadrants) and non-viable (positive 7-AAD staining, upper quadrants) cell populations and non-apoptotic (negative Annexin V, left quadrants) and apoptotic (positive Annexin V, right quadrants) were defined for the control cell population i.e. untreated, and used for all samples within the experiment. Boundaries are as follows:

7-AAD: 5.0×10^2

Annexin V: 2.2×10^4
