# Supplementary material for: Neurotoxic amyloidogenic peptides in the proteome of SARS-COV2: potential implications for neurological symptoms in COVID-19
Source: Nat Commun. 2022 Jun 13;13:3387. doi: 10.1038/s41467-022-30932-1 (PMC9189797; doi:10.1038/s41467-022-30932-1)
Supplement: Supplementary file 3 — Reporting Summary [file 41467_2022_30932_MOESM3_ESM.pdf]

nature portfolio

Corresponding author(s): Dr Nicholas Reynolds
Dr Joshua Berryman

Last updated by author(s): May 13, 2022

Reporting Summary

Nature Portfolio wishes to improve the reproducibility of the work that we publish. This form provides structure for consistency and transparency in reporting. For further information on Nature Portfolio policies, see our Editorial Policies and the Editorial Policy Checklist.

Statistics

For all statistical analyses, confirm that the following items are present in the figure legend, table legend, main text, or Methods section.

n/a	Confirmed	
☐	☒	The exact sample size (*n*) for each experimental group/condition, given as a discrete number and unit of measurement
☐	☒	A statement on whether measurements were taken from distinct samples or whether the same sample was measured repeatedly
☐	☒	The statistical test(s) used AND whether they are one- or two-sided<br>*Only common tests should be described solely by name; describe more complex techniques in the Methods section.*
☒	☐	A description of all covariates tested
☒	☐	A description of any assumptions or corrections, such as tests of normality and adjustment for multiple comparisons
☐	☒	A full description of the statistical parameters including central tendency (e.g. means) or other basic estimates (e.g. regression coefficient) AND variation (e.g. standard deviation) or associated estimates of uncertainty (e.g. confidence intervals)
☐	☒	For null hypothesis testing, the test statistic (e.g. *F*, *t*, *r*) with confidence intervals, effect sizes, degrees of freedom and *P* value noted<br>*Give P values as exact values whenever suitable.*
☒	☐	For Bayesian analysis, information on the choice of priors and Markov chain Monte Carlo settings
☒	☐	For hierarchical and complex designs, identification of the appropriate level for tests and full reporting of outcomes
☒	☐	Estimates of effect sizes (e.g. Cohen's *d*, Pearson's *r*), indicating how they were calculated

Our web collection on statistics for biologists contains articles on many of the points above.

Software and code

Policy information about availability of computer code

Data collection	Data Generation:<br>1. TANGO 2.2 Amyloid prediction web server tango.crg.es<br>2. ZIPPER version 1 - Amyloid prediction web server services.mbi.ucla.edu/zipperdb<br>3. NAB 1.3 Molecular geometry and construction tool ambermd.org<br>4. pmemd 19 Efficient time integrator ambermd.org
Data analysis	1. FiberApp version 1 freeware for the statistical analysis of amyloid nanofibrillar structures (available to download from the manucript webpage (https://pubs.acs.org/doi/abs/10.1021/ma502264c)<br>2. CRYSOL3 3.0.3 Orientationally averaged FFT of electron density www.embl-hamburg.de<br>3. pymol 2 Molecular visualisation pymol.org<br>4 Graph Pad Prism v8.4.2<br>5 Bruker Nanoscope Analysis v1.7<br>6 FloJo v.10.8.1<br>7 FACS Diva v.9

For manuscripts utilizing custom algorithms or software that are central to the research but not yet described in published literature, software must be made available to editors and reviewers. We strongly encourage code deposition in a community repository (e.g. GitHub). See the Nature Portfolio guidelines for submitting code & software for further information.

Data

Policy information about availability of data

All manuscripts must include a data availability statement. This statement should provide the following information, where applicable:

- Accession codes, unique identifiers, or web links for publicly available datasets
- A description of any restrictions on data availability
- For clinical datasets or third party data, please ensure that the statement adheres to our policy

The Authors declare that all the data supporting the finding of this study are available from the source data file in the supplementary information.

Field-specific reporting

Please select the one below that is the best fit for your research. If you are not sure, read the appropriate sections before making your selection.

[x] Life sciences [ ] Behavioural & social sciences [ ] Ecological, evolutionary & environmental sciences

For a reference copy of the document with all sections, see nature.com/documents/nr-reporting-summary-flat.pdf

Life sciences study design

All studies must disclose on these points even when the disclosure is negative.

Sample size	Initial peptide concentrations ranging from 5 mgmL-1 to 0.02 mgmL-1 covering 2 orders of magnitude. No specific statistical method was used to determine the range of concentrations studied. Analysis of the data revealed that at the lowest concentrations no significant differences in cell number of apoptosis were observed (figure 5) therefore we beleive this provides justification for the sample size chosen. Additionally the concentrations used cover the complete range of concentrations used to determine the cytotoxicity of other amyloid species in similar papers (i.e aBeta).
Data exclusions	No data was excluded
Replication	For the MTT assays the results shown are the mean of three independent experiments, all attempts at replication were successful. For the flow cytometery assays the results shown are the result of three independent experiments, the scatter plots (figure 5c) show representative results from one of these experiments, figure 5d shows the mean of these three independent experiments. For the flow cytometry assays all attempts at replication were successful. All experiments performed were performed as three independent experiments to confirm repeatability of these experiments, all attempts at replication were successful, many of the AFM experiments in this paper were reproduced in separate laboratories in Australia (La Trobe) and Switzerland (ETH).
Randomization	Automated analysis was performed for the MTT assay using a plate reader and the same gating strategy as the control samples were applied in flow cytometry analysis to avoid operator bias and consequently reduce the necessity of randomization of the samples. Further no randomization was performed for either the AFM analysis (FiberApp) or secondary structure analysis of the CD. However as this analysis is performed in a semi-automated manner by computer based algorithms we believe this has reduced the necessity of randomizing these samples.
Blinding	Automated analysis was performed for the MTT assay using a plate reader and the same gating strategy as the control samples were applied in flow cytometry analysis to avoid operator bias and consequently reduce the necessity of blinding of the samples. Further no blinding was performed for either the AFM analysis (FiberApp) or secondary structure analysis of the CD. However as this analysis is performed in a semi-automated manner by computer based algorithms we believe this has reduced the necessity of randomizing these samples.

Reporting for specific materials, systems and methods

We require information from authors about some types of materials, experimental systems and methods used in many studies. Here, indicate whether each material, system or method listed is relevant to your study. If you are not sure if a list item applies to your research, read the appropriate section before selecting a response.

Materials & experimental systems

n/a	Involved in the study	
[ ]	[x]	Antibodies
[ ]	[x]	Eukaryotic cell lines
[x]	[ ]	Palaeontology and archaeology
[x]	[ ]	Animals and other organisms
[x]	[ ]	Human research participants
[x]	[ ]	Clinical data
[x]	[ ]	Dual use research of concern

Methods

n/a	Involved in the study	
[x]	[ ]	ChIP-seq
[ ]	[x]	Flow cytometry
[x]	[ ]	MRI-based neuroimaging

Antibodies

Antibodies used	A11 antibody (Invitrogen, Product No: AHB0052, LOT:VF299837) & Goat-Anti Rabbit IgG-Alexa Fluor 647 (Lot 1871168 Product No A21244, Molecular Probes)
Validation	A11 antibody was confirmed for oligomer speficity against a positive control known to readily form oligomers (Phenylalanine assemblies).

Eukaryotic cell lines

Policy information about cell lines

Cell line source(s)	SH-SY5Y from ATCC
Authentication	None of the cell lines used were authenticated
Mycoplasma contamination	All cell lines used tested negative to microplasma infection
Commonly misidentified lines (See ICLAC register)	None of the cell lines used were authenticated

Flow Cytometry

Plots

Confirm that:

- [x] The axis labels state the marker and fluorochrome used (e.g. CD4-FITC).
- [x] The axis scales are clearly visible. Include numbers along axes only for bottom left plot of group (a 'group' is an analysis of identical markers).
- [x] All plots are contour plots with outliers or pseudocolor plots.
- [x] A numerical value for number of cells or percentage (with statistics) is provided.

Methodology

Sample preparation	SH-SY5Y cells from the ATCC were seeded onto 24 well plates at 1x105 cells per mL, and left to adhere for 24 hours. Peptide assemblies were added at a range of concentrations and incubated for 48 h. The cells were washed, enzymatically detached, resuspended in FACS buffer and transported to the flow cytometer on ice.
Instrument	FACS Aria III (BD Biosciences)
Software	Data Collection Software: BD FACSDiva™ Software<br>Analysis Software: FlowJo 10.8.1
Cell population abundance	Flow Cytometry was used for analysis only, no cell sorting was performed.
Gating strategy	Boundaries between viable (negative 7-AAD staining, lower quadrants) and non-viable (positive 7-AAD staining, upper quadrants) cell populations and non-apoptotic (negative Annexin V, left quadrants) and apoptotic (positive Annexin V, right quadrants) were defined for the control cell population i.e. untreated, and used for all samples within the experiment. Boundaries are as follows:<br>7-AAD: 5.0 x 102<br>Annexin V: 2.2 x 104

- [x] Tick this box to confirm that a figure exemplifying the gating strategy is provided in the Supplementary Information.
